# Bloodstream Infections Due to Carbapenemase-Producing *Escherichia coli*: A Comprehensive Review

**DOI:** 10.3390/antibiotics15020176

**Published:** 2026-02-05

**Authors:** Maria Scrascia, Adriana Antonina Tempesta, Viviana Cafiso, Carlo Pazzani, Maria Lina Mezzatesta

**Affiliations:** 1 Department of Biosciences, Biotechnology and Environment, University of Bari Aldo Moro, Via Orabona 4, 70125 Bari, Italy; maria.scrascia@uniba.it (M.S.); carlo.pazzani@uniba.it (C.P.); 2 Section of Microbiology, Department of Biomedical and Biotechnological Sciences, University of Catania, 95123 Catania, Italy; adriana.tempesta@unict.it (A.A.T.); vcafiso@unict.it (V.C.)

**Keywords:** carbapenemase-producing *Escherichia coli*, bloodstream infections, molecular epidemiology, NDM, OXA-48, KPC, IMP, horizontal gene transfer, plasmid-mediated resistance, high-risk clones

## Abstract

**Background/Objectives:** Carbapenemase-producing *Escherichia coli* (CP-Ec) has emerged as an important contributor to the global crisis of antimicrobial resistance. Although less prevalent than carbapenemase-producing *Klebsiella pneumoniae*, CP-Ec exhibits marked genomic plasticity, efficient plasmid-mediated dissemination, and increasing involvement in bloodstream infections. This comprehensive review summarizes the global epidemiology, molecular features, treatment options, clonal structure and transmission dynamics of CP-Ec. Particular attention is given to the expanding repertoire of NDM, OXA-48-like, and KPC carbapenemases and their associated plasmid backbones. Key high-risk clones, including ST410, ST167 and ST131, are highlighted as drivers of international spread. **Conclusions and Future Directions:** CP-Ec bloodstream infections represent a growing clinical challenge, often associated with severe outcomes and limited therapeutic options, particularly for NDM producers. The emergence of treatment failures with last-resort agents further underscores the need for improved management strategies. Strengthened global surveillance, integration of genomic epidemiology, optimized antimicrobial stewardship, and targeted infection control measures are essential to limit the dissemination of CP-Ec and mitigate its impact on human health.

## 1. Introduction

Antimicrobial resistance (AMR) has emerged as a critical global health challenge. Resistant microorganisms circulate through interlinked reservoirs—including water, soil, healthcare facilities, agricultural settings, and the broader community—underscoring the interconnected nature of human, animal, and environmental health systems [1]. In recognition of this complexity, major international organizations (Food and Agriculture Organization of the United Nations—FAO; the United Nations Environment Programme—UNEP; the World Organization for Animal Health—OIE and the World Health Organization—WHO) encouraged addressing this issue by a One Health approach, which integrates molecular epidemiology, ecological dynamics, and host–pathogen interactions to better understand the evolution and dissemination of AMR [2].

In clinical settings, AMR is a major concern. The rise in multidrug-resistant (MDR) bacteria—defined as resistance to three or more antibiotic classes—has compromised commonly used therapies and increased infection-related mortality. A global analysis estimated that by 2050, MDR organisms may account for up to 10 million deaths annually [3]. Healthcare-acquired infections (HAIs)—including respiratory tract infections, surgical site infections, urinary tract infections, gastrointestinal infections, and bloodstream infections (BSIs)—are among the leading adverse events in hospitalized patients. BSIs, in particular, represent the third leading cause of mortality in Europe [4] and were designated a Global Health Threat by the WHO in 2017 [5] especially when caused by MDR organisms [6].

Gram-negative pathogens, especially *Klebsiella pneumoniae* and *Escherichia coli*, are among the principal causes of BSIs [7,8]. Although *E. coli* has historically been less frequently implicated in BSIs than *K. pneumoniae* [9] it accounted for the highest number of deaths attributable to MDR organisms in 2019 [3]. Of particular concern is the growing resistance to carbapenems that are considered the most reliable resort against bacterial infections [7,10]. In this context, carbapenem-resistant *E. coli* (CREC) pose a major challenge due to their high-level resistance and expanding presence in hospitals, communities, maternal–neonatal networks, and environmental reservoirs [11].

Carbapenemase-producing *E. coli* (CP-Ec) exhibits diverse resistance mechanisms and frequently carry multiple β-lactamases within MDR or extensively drug-resistant (XDR) genomic backgrounds. The global rise in CREC correlates closely with the dissemination of NDM and OXA-48-like enzymes [12], plasmid mobility, and expansion of internationally recognized clones such as ST410, ST131, ST167, and ST405 [10]. The spread of carbapenemase genes is largely driven by mobile genetic elements—including transposons, integrons, and highly transmissible plasmids—that facilitate rapid horizontal transfer across species and settings [13]. Together, gene mobility, limited treatment options, and food safety concerns make CREC infections a major public health [11,14]. Despite this emerging threat, studies specifically examining the detection and genomic localization of carbapenemase genes in CREC bloodstream isolates remain limited. While a recent evaluation examined outcomes associated with group D carbapenemase-producing *Enterobacterales* bacteremia [15], no comprehensive reviews focused on carbapenemase-producing *E. coli* BSIs have been published in the last decade.

The present review addresses this gap by summarizing global trends in carbapenemase genes among CREC BSI isolates, with particular emphasis on the geographical distribution of strains and the genomic localization of genes.

Specifically, the objectives of this review are to (i) characterize the diversity and distribution of carbapenemase genes in BSI isolates; (ii) examine the relationship between carbapenemase variants, plasmid -Inc groups, sequence types (STs), and internationally recognized high-risk clones; (iii) describe the clinical characteristics of affected patient populations, including adult, pediatric, and neonatal settings; (iv) summarize available therapeutic options and emerging challenges in the management of CP-Ec. Together, these aims provide a comprehensive, clinically oriented overview of the molecular epidemiology, pathogenic potential, and therapeutic implications of CP-Ec BSIs.

## 2. Methods

### 2.1. Search Strategy and Selection Criteria

**Figure 1:**
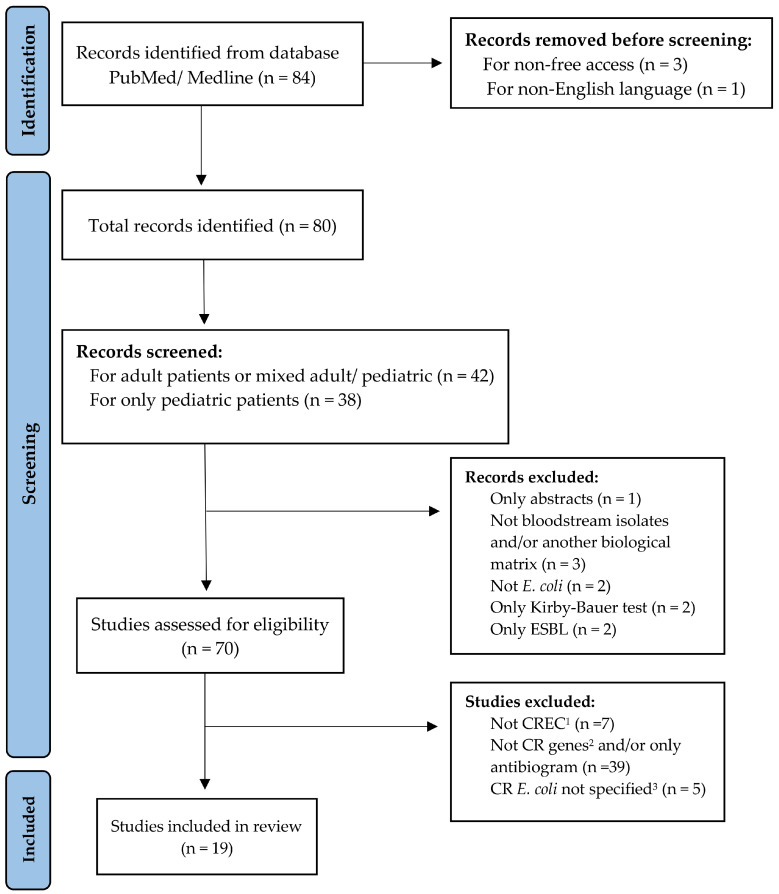
PRISMA-style flow diagram illustrating the identification, screening, eligibility assessment, and inclusion of studies reporting CP-Ec BSI isolates. **Not CREC^1^** = *E. coli* not resistant to carbapenems. **Not CR genes^2^** = Carbapenem resistance genes not detected. **CR *E. coli* not specified^3^** = Cases where *E. coli* identification was uncertain or not clearly differentiated from other pathogens.

A comprehensive search among accessible publications was conducted to select studies reporting bloodstream infections caused by CP-Ec worldwide. The literature was retrieved from PubMed/MEDLINE using the terms (“neonates”, OR “pediatrics”, OR “adults”) AND “*Escherichia coli*” AND “bloodstream infections” AND (“carbapenemase genes” OR “carbapenem”). Eligible study designs included case reports, case–control studies, genomic notes, and retrospective or prospective observational studies. The search was limited to the last decade (2015–2025) and yielded 84 records. Studies were screened to identify reports of BSI isolates with confirmed carbapenem resistance genes and, when available, information on their genomic localization. As this is a comprehensive review rather than a formal systematic review with meta-analysis, we did not apply a structured risk-of-bias tool. Instead, each article was qualitatively assessed with attention to methodological rigor, transparency of outcome reporting, and potential sources of confounding. For transparency, the study selection process is summarized in a PRISMA-style flow diagram (Figure 1) [16].No protocol was registered (e.g., PROSPERO).

### 2.2. Inclusion and Exclusion Criteria

Studies were included if they reported CP-Ec bloodstream infections with available molecular data. All carbapenem-resistant clinical isolates, included in this study, were analyzed to determine the presence of carbapenemase-encoding genes using molecular methods, including polymerase chain reaction (PCR) analyses and sequencing or Whole Genome Sequencing (WGS) on the Illumina platform. Furthermore, in several included studies, carbapenemase detection also relied on screening tests, including phenotypic assays (e.g., mCIM/eCIM, EDTA-based synergy testing, and the modified Hodge test) as well as rapid immunochromatographic (lateral flow) immunoassays. We excluded studies involving non-BSI samples, non-*E. coli* isolates, non-carbapenem-resistant strains, absence of carbapenemase gene detection, ESBL-only producers, or publications not available in English or open access. This open-access restriction may have limited retrieval of some relevant evidence.

### 2.3. Study Selection and Data Extraction

**Figure 2:**
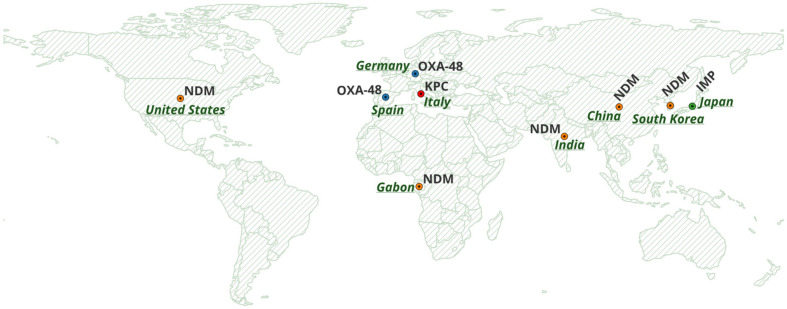
Global distribution of carbapenemase genes (NDM, OXA-48, KPC and IMP). Basemap created with OpenStreetMap data (© OpenStreetMap contributors).

All retrieved records were screened by three investigators, who independently evaluated titles, abstracts, and full texts. Discrepancies were resolved by consensus. Extracted data included country of origin, study design, patient population, number of carbapenem-resistant isolates, sequence types, carbapenemase genes, additional resistance determinants, and genomic localisation when available. When reported, plasmid characterization was mainly based on in silico WGS approaches (e.g., PlasmidFinder/pMLST) and, in some cases, conjugation assays to assess transferability. Only studies achieving full agreement among reviewers were included in the final dataset. Geographic distribution maps (Figure 2) were produced using QGIS (v3.44.5 ‘Solothurn’; https://qgis.org; accessed on 1 February 2026).

## 3. Global Epidemiology and Molecular Characteristics

The global distribution of CP-Ec causing BSIs reflects a complex interplay between regional epidemiology, resistance mechanisms, clonal lineages, and plasmid-mediated transfer of carbapenemase genes. Although still less prevalent than carbapenemase- producing *Klebsiella pneumoniae*, CP-Ec exhibits wide genetic heterogeneity and a strong reliance on plasmid-mediated transfer, resulting in geographically distinct resistance landscapes. Table 1 provides an integrated overview of the clinical, and molecular and genomic findings of CP-Ec bloodstream isolates across countries, summarizing the data reported in the included studies.

### 3.1. Europe

#### 3.1.1. Spain: Plasmid-Mediated OXA-48 Dissemination in a Low-Burden Setting

In the CARB-ES-19 national survey [17] conducted across all Spanish provinces, CP-Ec represented only a small fraction of carbapenemase-producing *Enterobacterales* (CPE), accounting for 6.5% of all CPE detected. Their overall prevalence among more than 70,000 *E. coli* isolates was extremely low (0.04%), and bloodstream infections were particularly uncommon, with only two CP-Ec isolates recovered from blood (0.06%). OXA-48 was the predominant carbapenemase (73.1%), followed by VIM-1, KPC-3, and less frequently, NDM-5, confirming the strong dominance of OXA-48-like enzymes on the Iberian Peninsula. Co-production of carbapenemases was rare, although one isolate carried both *bla*_OXA-48_ and *bla*_VIM-1_. Genotypic analysis demonstrated marked polyclonality, with 21 distinct sequence types (STs) identified and most represented by a single isolate. ST131 was the only lineage detected more than once and the only one associated with more than one carbapenemase type, although it did not exhibit the expansion patterns characteristic of high-risk *K. pneumoniae* clones circulating in the same dataset. Overall, these findings, together with evidence from other studies [36], suggest that CP-Ec dissemination is polyclonal and primarily driven by the spread of conjugative plasmids that can mobilize different carbapenemase genes.

#### 3.1.2. Italy: A KPC-Endemic Environment with Sporadic Alternative Carbapenemases

A large multicenter surveillance study comprising 142 bloodstream infections demonstrated that CP-Ec constituted only a minority of carbapenemase-producing *Enterobacterales*, yet their detection was associated with significant clinical impact across intensive care, medical, and surgical wards [18]. KPC was the predominant carbapenemase, with only sporadic detection of VIM, NDM and OXA-48. The study did not systematically include MLST or genomic localisation of the carbapenemase genes, limiting the reconstruction of clonal relationships, although the enzyme distribution closely mirrors the established KPC-driven epidemiology of the Italian healthcare system.

#### 3.1.3. Germany: Multidrug-Resistant OXA-48 Producers Driven by IncL Plasmids

In Germany, two OXA-48-producing *E. coli* bloodstream isolates were reported, belonging to ST393 and ST354 [19]. Both strains carried CTX-M-type ESBLs and a broad repertoire of resistance determinants, including aminoglycoside-, macrolide-, sulfonamide- and tetracycline-resistance genes, indicating extensive multidrug-resistance. Genomic analysis revealed the association of *bla*_OXA48_ to both the epidemic IncL and IncF plasmids, underscoring the central role of IncL in dissemination of OXA-48 across European *Enterobacterales* populations. Across Europe, CP-Ec remain relatively uncommon, with OXA-48-like enzymes representing the dominant resistance mechanism and largely disseminated via the epidemic IncL plasmid. The pronounced genetic heterogeneity of circulating isolates underscores the great contribution of plasmid transfer in addition to the single ST clone expansion.

### 3.2. Asia

#### 3.2.1. China: Extensive NDM Dissemination Sustained by High-Risk Clones and IncX3 Plasmids

In Asia, most of the available data on CP-Ec BSIs comes from China and consistently points to a scenario dominated by NDM-producing lineages. One of the earliest descriptions reported three adult hematology patients infected with ST167 isolates carrying the *bla*_NDM-5_ gene, marking the initial recognition of the ST167–NDM-5 combination in BSIs [20]. In a previous multicentre ICU and NICU cohort, three NDM-1 producing isolates belonging to ST361, ST40 and ST410 were identified, already indicating early clonal heterogeneity and the presence of ST410 as an emerging lineage in the Chinese setting [21]. More recent hematology cohorts have confirmed the persistence and expansion of NDM-producing *E. coli* in high-risk adult populations over several years, although molecular typing and plasmid characterization were not systematically performed [22].

Genome-based investigations from China have substantially refined the epidemiological understanding of CP-Ec, highlighting a scenario characterized by marked clonal diversity and sustained circulation of carbapenem-resistant lineages across different clinical settings. Early genomic reports already indicated the presence of heterogeneous CP-Ec populations within BSIs [23,24,25] suggesting that resistance has emerged multiple times and through distinct evolutionary pathways rather than from the expansion of a single epidemic clone.

These strains commonly co-harbor multiple β-lactamases (CTX-M variants, CMY-2/30, TEM-1B, OXA-1). Their resistome is broad and includes aminoglycoside-modifying enzymes, plasmid-mediated fluoroquinolone resistance, and fosfomycin resistance. Additional resistance strains include macrolide and tetracycline genes, as well as chromosomal mutations affecting quinolone targets. Pediatric cohorts mirror adult findings, showing high heterogeneityand recurrent involvement of ST410 and ST131, with the *bla*_NDM-5_ gene frequently located on IncX3 or multireplicon IncF plasmids [26]. A multicenter survey including both adult and pediatric patients similarly reported broad heterogeneity across hospitals and regions and highlighted IncX3 dissemination as a key driver [27]. Collectively, these genomic and multicenter studies indicate that CP-Ec in China circulates through both clonal spread and plasmid-mediated transmission, contributing to a dynamic and firmly established reservoir of CP-Ec within high-risk clinical environments.

#### 3.2.2. South Korea: A Mosaic Carbapenemase Landscape with Coexisting Mechanisms

Outside China, available data remain more limited but indicate a heterogeneous epidemiological scenario. In South Korea, national surveillance identified bloodstream isolates producing KPC-2, the rare KPC-18 variant, NDM-5 and OXA-181, frequently accompanied by CTX-M-type ESBLs and AmpC enzymes such as DHA-1 and CMY-2 [28]. Taken together, these observations contrast sharply with the NDM-dominated epidemiology documented in China and illustrate a more mosaic distribution of carbapenemases across the region.

Overall, the Asian landscape of CP-Ec is marked by the overwhelming predominance of NDM-producing lineages in China—driven by possible widespread dissemination of IncX3 plasmids and the expansion of high-risk clones such as ST410, ST167 and ST131 [20,21,26,27]—while neighboring countries display more diverse combinations of KPC, OXA-181 and NDM enzymes [28]. This pattern underscores a dynamic regional ecology shaped simultaneously by clonal spread and frequent plasmid-mediated gene transfer, reinforcing Asia’s central role as a major global reservoir for CP-Ec.

#### 3.2.3. Japan: IMP-6 Circulation Driven by Broad-Host-Range IncN Plasmids

Available data from Japan, though limited, provide important insights into the regional epidemiology of CP-Ec and highlight the distinctive role of IMP-type metallo-β-lactamases in this setting. A retrospective case–control study conducted between 2008 and 2013 identified three adult bloodstream infections caused by *E. coli* producing IMP-6, belonging to ST131 (two isolates) and the rare ST2750 lineage [29]. All isolates also carried CTX-M-type ESBLs, mainly CTX-M-2 and CTX-M-27, illustrating the coexistence of carbapenemase production with additional β-lactam-resistance determinants. The predominance of ST131 among these early cases underscores the capacity of globally disseminated pandemic clones to act as vehicles for region-specific carbapenemase genes.

A subsequent investigation, conducted in 2013–2014, described a further IMP-6-producing ST131 bloodstream isolate harboring a conjugative IncN plasmid [30]. This study formed part of a broader analysis documenting a prolonged multispecies outbreak in Japan, driven not by clonal expansion but by efficient plasmid-mediated transmission of *bla*_IMP-6_. The identification of a type A1 IncN plasmid with an intact integron structure highlighted the broad host range of these plasmids and their capacity to spread carbapenemase genes across different *Enterobacterales* species and clinical wards.

Together, these findings indicate that the Japanese epidemiology of CP-Ec is shaped primarily by the horizontal dissemination of IMP-6 via IncN plasmids, often involving the pandemic ST131 lineage. Unlike the NDM-dominated patterns observed in China or the mixed carbapenemase landscape reported in South Korea, the Japanese scenario appears more plasmid-driven and IMP-centric, underscoring the regional distinctiveness of carbapenem resistance mechanisms in East Asia.

#### 3.2.4. India: Pediatric NDM Producers and Emerging Mixed Carbapenemase Profiles

Although fewer data are available, evidence from India highlights the clinical relevance of CP-Ec in pediatric settings. In a retrospective cohort (2014–2015), *bla*_NDM_ gene was detected in 8 of 11 bloodstream isolates, with co-production of NDM and an OXA-48-like enzyme in one isolate [31]. Two carbapenem-resistant isolates carried no detectable carbapenemase gene, suggesting alternative mechanisms such as ESBL or AmpC production with porin alterations. While STs and plasmid structures were not provided, the predominance of NDM and the presence of NDM–OXA-48 co-producers place India within the broader South Asian reservoir of NDM-producing *E. coli*.

### 3.3. Africa (Central Africa: Gabon)

In Africa, the epidemiology of CP-Ec remains poorly characterized, with only limited data available. However, emerging evidence indicates that clinically significant resistance mechanisms are already present across both hospital and community settings. A recent prospective cohort from Gabon reported a pediatric bloodstream infection caused by an NDM-5-producing *E. coli* belonging to the uncommon ST2083 lineage [32]. The detection of this rare sequence type in a neonatal clinical context illustrates that novel or previously under-recognized clones may be circulating in the region.

Within the same study, maternal and neonatal colonization by NDM- and OXA-48-producing *Enterobacterales* was also documented, raising concern for vertical transmission and early-life acquisition of multidrug-resistant organisms. Although the available data remain limited, these findings suggest that mechanisms driving carbapenem resistance globally—particularly the dissemination of NDM-5 and the spread of highly mobile plasmids such as IncX3—are also emerging in Central Africa. This highlights the potential for the continent to act as an evolving reservoir of carbapenemase producers in settings where microbiological surveillance is still insufficiently implemented.

### 3.4. North America (United States)

In the United States, CP-Ec BSIs remain relatively rare but increasingly associated with NDM-producing strains, often linked to travel or importation. A recent case report documented three consecutive NDM-5-producing isolates from a single pediatric patient of Indian origin, demonstrating both international introduction and intra-host microevolution [33]. A larger retrospective cohort of 28 BSI cases revealed remarkable clonal heterogeneity (multiple STs including ST2, ST692, ST53, ST87, ST88, ST960, ST8, ST1017, ST477, ST39) and a predominance of NDM-5, followed by NDM-1 and sporadic KPC-2 producers [34]. Taken together, these observations suggest that the current U.S. scenario may reflect polyclonal, plasmid-mediated introductions of NDM-producing strains rather than the expansion of established endemic clones.

### 3.5. Global Observations

Across regions, the epidemiology of CP-Ec BSIs is shaped by a combination of horizontal gene transfer mediated by mobile plasmids and region-specific carbapenemase pressures. As summarized in Figure 2, based on the included studies, the global distribution of major carbapenemase families, including NDM, OXA-48-like, KPC, and IMP, illustrates heterogeneous geographic patterns associated with distinct resistance determinants. In the European context, the number of available studies was limited. The included studies were conducted exclusively in Germany, Italy, and Spain, as no other European country met the inclusion criteria. In these countries, the distribution of carbapenemases is largely characterized by OXA-48-like enzymes frequently associated with IncL plasmids, whereas China shows sustained NDM-driven circulation supported by IncX3 plasmids and high-risk lineages; Japan represents a distinct IMP-6/IncN plasmid–centered scenario. Emerging data from Africa and North America remain sparse, underscoring the need for strengthened surveillance to better define local reservoirs and transmission dynamics. Evidence from multispecies outbreaks [30] and neonatal colonization clusters [32] underscores the central role of plasmid-mediated transfer in driving global dissemination, often exceeding the impact of clonal spread.

## 4. Association Between Carbapenemase Genes and Plasmids

Role of inter- and intra-species horizontal gene transfer in the dissemination of antimicrobial resistance is highlighted in an ever-increasing number of studies focusing on genes encoding for carbapenemases in *Enterobacterales*. In this paragraph, we focused, among the CP-Ec strains reported in the selected papers, on the subset for which data regarding plasmid localization and/or transfer of detected carbapenemase genes are reported (Table 2).

Indeed, eight papers published data on plasmids harboring carbapenemase genes in a total of 22 CP-Ec strains. Nineteen of them have been isolated in China during a period ranging from 2016 to 2020 and harbored three different NDM variants (NDM-1, NDM-3, NDM-5) associated with different plasmids [23,24,25,26,27,35]. In more detail, Li and colleagues [23] reported the draft genome of the first NDM-3 producing *E.coli* strain (ST977) isolated from BSI (in 2018) and the plasmid localization of the gene. The Inc group of the plasmid (ca. 150 kb length) has been assigned to IncFI1 or F1B or F1A, being those detected in the draft genome. However, no experimental data are reported about the possible transmissibility of the plasmids and on which plasmid the gene is harbored. The study is the only one, among the selected references, referring to the gene for NDM-3.

In the case of the most frequently detected variant NDM-5, 5 papers out 8 showed the association of the gene to different plasmids [24,25,26,27,35]. The strains belonged to different STs and most of them harbor the *bla*_NDM-5_ gene localized on a IncX3 plasmid [24,25,27,35]. In the remaining strains, the gene is associated with a IncFII plasmid (2 strains), IncI (1 strain), IncFII/I1 (1 strain) or InFIA/FIB/FII/Q1 (3 strains) plasmids [26,27]. The paper of Huang and colleagues [26] is the only one in which the transfer of the *bla*_NDM-5_ (frequency ranging from 2.5 × 10^−3^ to 3.5 × 10^−6^) mediated by the IncX3 and IncFII/I1 plasmids has been demonstrated in four strains. The authors emphasize how IncX3 plasmid is a key element in disseminating *bla*_NDM-5_ among *E. coli* and other species. Although publications on association between NDM variants and plasmids are prevalent among the selected papers, two of them reported the detection of OXA-48 and IMP-6 carbapenemase genes, which were transferable via IncL, IncF or IncN plasmids [19,30]. The study from Hamprecht and colleagues reports the plasmid-mediated transfer of *bla*_OXA-48_ from 2 BSI CREC strains (ST393 and ST 354) isolated in Germany during 2010–2017. *bla*_OXA-48_ is on a 63 kb IncL plasmid in 1 strain and on 80 kb IncF plasmid in the other one. Both the plasmids were conjugative, and frequencies are 1 × 10^−1^ and ca. 2 × 10^−7^, respectively. The IncL plasmid showed a broad host range transfer in *Enterobacterales* by disseminating worldwide with more efficient transfer compared to the IncF. The authors speculate that these properties might lead to persistence of *bla*_OXA-48_ in environmental niches, healthy humans and animals without antibiotic pressure, contributing to further dissemination. About IMP-6 carbapenemase-producing *E. coli* from BSIs, the study published by Yamagishi et al. in 2020 refers to one strain isolated from Japan in 2013 [30]. The isolate belonged to the ST131 and harbored a IncN conjugative plasmid characterized by an intact *IntI1* structure. It is known that the IncN plasmid belongs to a broad-host-range group of plasmids possibly responsible for the multispecies outbreaks of *bla*_IMP_ carrying Gram-negative pathogens. A regular association between the strain STs and specific carbapenemase genes did not emerge, suggesting a broader contribution of plasmids in the dissemination of carbapenem resistance. Considering the limited number of papers focusing on genomic localization of carbapenemase genes, we cannot speculate any correlation among those genes and Inc group in CRECs. However, it is possible to note that the IncX3 was the predominant plasmid type observed, being reported as the most prevalent vehicle for *bla*_NDM-5_ and its variants. In this point of view, it should be noted the importance of plasmid epidemiological analyses in providing clues to control outbreaks from carbapenemase-producing *Enterobacterales* in healthcare settings and to avoid diffusion of carbapenem-resistance in animals and environment in the context of the One Health approach.

## 5. STs and High-Risk Clones

Across the 19 included studies—conducted in nine countries and encompassing adult and neonatal populations—the distribution of *E. coli* STs associated with CP-Ec displayed extensive genetic heterogeneity. ST information was available in 14 of the studies and revealed that no single clonal lineage dominates globally; rather, CP-Ec BSIs arise from a diverse pool of strains that vary considerably by region, setting, and associated resistance mechanisms.

Among the detected clones, ST410, ST131, and ST167 emerged as the most recurrent In more detail, ST410 was consistently reported across multiple Chinese cohorts [21,24,26,27,35], supporting its status as one of the most successful high-risk global lineages and one that is frequently associated with *bla*_NDM-5_. ST167, another important lineage within the *E. coli* phylogeny, was reported mainly in two independent Chinese studies [20,27] and showed strong associations with both NDM enzymes and co-carried ESBLs. ST131, the well-recognized pandemic clone, was identified across Asia and Europe [17,26,29,30]. Although traditionally linked to CTX-M-type ESBLs, these data highlight its growing involvement in carbapenemase dissemination, underscoring its adaptability and epidemic potential.

Additional recurrent clones such as ST405, ST361, and ST354 were identified in several studies, while a range of uncommon or previously unreported STs—including ST2083 in Gabon [32] and ST977 in China [23]—illustrate the expanding clonal diversity of CP-Ec BSIs. Large multicenter and longitudinal investigations [34] further confirmed the polyclonal nature of CREC circulation, documenting numerous distinct STs within single study periods and healthcare networks.

Taken together, these findings reveal a globally heterogeneous clonal landscape, where well-recognized high-risk lineages—including ST410, ST167 and ST131—coexist with a wide array of sporadic or emerging clones. This pattern suggests that the epidemiology of CP-Ec BSIs is driven not by the expansion of a single dominant lineage, but rather by the convergent acquisition of carbapenemase genes across multiple genetic backgrounds, facilitated by highly mobile plasmids and region-specific selective pressures.

## 6. Clinical Characteristics of CP-Ec BSIs

### 6.1. Adult Populations

In adult patients, CP-Ec BSIs arise predominantly from urinary tract infections, intra-abdominal infections, and catheter-associated sources. Across the available studies, these infections consistently presented with severe clinical manifestations, frequently progressing to sepsis or septic shock. Comparative analyses suggest that while carbapenemase-producing *K. pneumoniae* continues to account for a higher burden of mortality in many settings, CP-Ec BSIs nevertheless carry substantial clinical risk and are associated with significant morbidity [22]. The convergence of multiple healthcare-associated risk factors—such as invasive devices, prolonged hospitalization, and recent antibiotic exposure—together with frequent delays in initiating appropriate antimicrobial therapy, substantially contributes to the clinical severity of CP-Ec BSIs.

### 6.2. Pediatric Populations

Pediatric cases of CP-Ec BSIs, although less common, exhibit a similarly severe clinical profile. Most reported episodes involve NDM-5–producing strains, particularly in neonates and immunocompromised children. Clinical presentations are frequently severe at onset, often requiring escalation to intensive care support [14,34]. In several cohorts, CP-Ec infections emerged in vulnerable patients with underlying hematologic or neonatal conditions, suggesting a combination of host factors and healthcare-associated exposures plays a critical role in facilitating invasive disease.

### 6.3. Community-Acquired CP-Ec BSIs

Community-acquired CP-Ec BSIs remain rare but documented. One of the clearest examples derives from a Chinese case of an NDM-5–producing ST410 strain causing bloodstream infection in a patient without recent healthcare exposure [35]. Such reports highlight the potential for globally disseminated high-risk lineages, particularly ST410, to establish reservoirs outside hospital environments, raising concerns regarding broader community transmission pathways. While the overall contribution of community acquisition remains limited, these observations underscore the importance of continued surveillance beyond traditional healthcare settings.

## 7. Therapeutic Challenges and Outcomes

CP-Ec BSIs represent a therapeutic challenge because they occur mainly in highly exposed, clinically fragile patients and often present as sepsis or septic shock, leaving little margin for delays or suboptimal early therapy [20,30,37,38,39]. The highest-risk groups include neonates and hematologic patients, frequently affected by NDM-producing strains in intensive-care environments [26,40,41]. This vulnerability is consistent with ExPEC biology, as these lineages preferentially cause invasive disease in hosts with compromised barriers or immunity [42].

From a clinical standpoint, the marked geographic heterogeneity in carbapenemase epidemiology—including the broad dissemination of OXA-48-like enzymes in several European settings—supports a mechanism-informed approach to therapy and prevention [43]. The dissemination of epidemic resistance plasmids and high-risk clones across settings, as described for multidrug-resistant *Enterobacterales*, provides a key framework to interpret CP-Ec spread and outbreak potential [44]. In parallel, the broader epidemiology of β-lactamase-producing pathogens (including ESBL and AmpC producers) and fluoroquinolone or aminoglycoside-modifying enzymes, helps explain why CP-Ec BSIs often present with extensive co-resistance that limits therapeutic options [44,45,46].

Consistent with this clinical complexity, across the included studies, outcomes were most often reported as crude in-hospital or 14–30-day mortality [8]. Attributable mortality was rarely estimated, as most CP-Ec BSI reports were retrospective and descriptive. However, studies applying adjustment methods in nosocomial BSI cohorts suggest excess mortality related to the infection and to delays in appropriate initial therapy, which are more likely in MDR settings [47,48,49,50].

### Clinical and Therapeutic Implications

The clinical complexity posed by CPE les has been well documented, with persistent gaps in effective treatment strategies even as newer agents become available [51]. Therapeutic options should be interpreted in relation to the carbapenemase type [52].

KPC-producing isolates (serine carbapenemases) typically show broad β-lactam resistance, and treatment commonly relies on newer BL/BLI agents active against KPC (e.g., ceftazidime–avibactam, meropenem–vaborbactam, imipenem–relebactam) [52].

In contrast, OXA-48-like producers may display lower-level carbapenem hydrolysis and can test susceptible to imipenem/meropenem. Moreover, OXA-48-like enzymes have limited activity against expanded-spectrum cephalosporins, so susceptibility may be preserved when ESBL/AmpC are absent [43,52]. Accordingly, when in vitro testing confirms susceptibility, carbapenems may still be considered as targeted therapy in selected OXA-48-like scenarios [52]. 

Metallo-β-lactamase (MBL) producers (e.g., NDM/VIM/IMP) remain the greatest therapeutic challenge because most BL/BLI combinations are inactive. Aztreonam is intrinsically stable to MBLs but can be inactivated by co-produced serine β-lactamases, providing the rationale for combining aztreonam with ceftazidime–avibactam. Consistently, the IDSA AMR guidance recommends ceftazidime–avibactam plus aztreonam (or cefiderocol) as key options for severe infections caused by MBL-producing *Enterobacterales*, with practical dosing suggestions including concurrent administration to optimize exposure [52]. Cefiderocol represents one of the few agents with reliable in vitro and clinical activity against NDM-producing *Enterobacterales* due to its siderophore-mediated uptake and stability against hydrolysis by metallo-β-lactamases [53].

Nonetheless, outcomes can be undermined by resistance emergence and by the accumulation of multiple mechanisms as follows: resistance to ceftazidime–avibactam has been documented to emerge during therapy in KPC-producing *Enterobacterales* [54,55], and cefiderocol failures in NDM-producing *E. coli* BSIs suggest selection of additional determinants under treatment pressure [33]. Importantly, despite this variability, reports of multi-mechanism carbapenemase-producing *Enterobacterales*—where combined β-lactamase backgrounds and porin alterations compromise multiple newer BL/BLI agents—suggest cefiderocol may still represent a salvage option in selected cases [56].

This reinforces the importance of rapid molecular diagnostics and, in selected cases, genomic approaches (e.g., WGS/NGS) to resolve complex multi-mechanism resistance, including porin alterations, and guide early, targeted therapy [40]. Lastly, access to newer agents and combination strategies may vary across countries due to formulary restrictions, reimbursement pathways, and acquisition costs, underscoring the need for locally adapted treatment algorithms.

## 8. Conclusions and Future Directions

CP-Ec is an emerging yet increasingly consequential cause of BSIs. The extensive co-resistance profiles characteristic of these isolates markedly restrict therapeutic options and frequently lead to delays in the initiation of effective treatment. Early molecular identification, informed empirical therapy, and robust infection-control measures therefore remain essential components in the clinical management of these infections.

Containment requires distinguishing clonal transmission from horizontal dissemination of carbapenemase genes. Clonal expansion is typically consistent with patient-to-patient spread and lapses in hospital hygiene measures, thus calling for strengthened IPC (screening, cohorting/isolation, hand hygiene). Conversely, the spread of carbapenemase genes among unrelated lineages is promoted by antibiotic selection pressure, supporting antimicrobial stewardship to minimize unnecessary broad-spectrum exposure. Continued genomic surveillance and coordinated IPC–stewardship strategies will be critical to limiting further dissemination and mitigating the growing clinical impact of CP-Ec BSIs.

## Figures and Tables

**Table 1:** Studies reporting CP-Ec bloodstream infections (BSIs): country and clinical setting, patient population, sequence types, carbapenemase and additional antimicrobial resistance determinants by class, with corresponding references.

Country	Pediatric or Adult Patient	Ward	No. of CP-Ec from BSIs	STs	CPase Genes	ESBL Genes	Amino-R Genes	Qn-R Genes	Mac-R, Tet-R, Sul-R and Other Genes	Reference
**Spain**	Adult	NR^1^	2	ST131, Other ST	*bla*_OXA-48_ *bla*_VIM-1_, *bla*_KPC-3_ *bla*_NDM-5_	*bla*_CTX-M-15_	NR^1^	*qnrB1-like*	*mcr-1*, *mcr-9* *tetA*, *sul1*, *sul2* *dfrA14*, *catB3*	[17]
**Italy**	Adult and Pediatric	ICU^2^, General medicine Surgical	142	NR^1^	*bla*_KPC_, *bla*_NDM_ *bla*_OXA-48_ *bla*_VIM-1_	NR^1^	NR^1^	NR^1^	NR^1^	[18]
**Germany**	Adult	Tertiary care hospitals	2	ST393 ST354	*bla*_OXA-48_	*bla*_CTX-M-1_, _-24_	*aadA5*, *aph(3″)-Ib*, *aph(6)-Id*	NR^1^	*tet(34)*, *tet(B)* *sul1*, *sul2*, *dfrA17*, *mph(A)*	[19]
**China**	Adult	Hematology	3	ST167	*bla*_NDM-5_	NR^1^	NR^1^	NR^1^	NR^1^	[20]
**China**	Adult and Pediatric	ICU^2^, NICU^3^	3	ST361, ST40 ST410	*bla*_NDM-1_	*bla*_TEM-1_ *bla*_CMY-30_ *bla*_CTX-M-15_, _-55_	RmtB	NR^1^	*fos*A3	[21]
**China**	Adult	Hematology	45	NR^1^	*bla*_NDM_ Other MBL Ser-CPase	NR^1^	NR^1^	NR^1^	NR^1^	[22]
**China**	Adult	NR^1^	1	ST977	*bla*_NDM-3_	*bla*_TEM-1B_ *bla*_OXA-1_ *bla*_CTX-M-55_	*aadA5*, *aac(3)-IId*, *aac(60)-Ib-cr*, *aa(3)-Iia*, *aadA2*	aac(6′)-Ib-cr	*mph(A)*, *tet (B)*, *sul1*, *dfrA12*, *dfrA17*, *catB4*, *RND*, *MFS*, *astA*, *fimABCDEFGHI*, *pilABC*, *capU iha*,	[23]
**China**	Adult	NR^1^	1	ST410	*bla*_NDM-5_	*bla*_CTX-M-15_ *bla*_CTY-2_ *bla*_TEM-1B_ *bla*_OXA-1_	*aac(3)-Iia, aac(6′)-Ib-r*, *aph(6)-Id*, *aph(3″)-Ib*, *aadA5*	aac (6′)-Ib-cr	*mph(A)*, *tetB*, *sul1*, *sul2*, *dfrA17*, *catB3*, *mdf(A)*, *ipfA*, *yidE*	[24]
**China**	Adult	NR^1^	1	ST354	*bla*_NDM-5_	*bla*_CTX-M-14_ *bla*_TEM-1B_	NR^1^	NR^1^	*tet(B)*, *mdf(A)*	[25]
**China**	Pediatric	Hematology, Neonatology Gastro- enterology	8	ST38, ST58, ST131, ST156 ST661, ST410	*bla*_NDM-5_	*bla*_CTX-M-14_, _-15_, _-65_ *bla*_CMY-2_ *bla*_TEM-1B_ *bla*_OXA-1_	*aac(3)-IVa* *aac(6′)Ib-cr*, *strA*, *strB, aadA2*, *aadA5* *aph(3′)-Ia*, *aph(4)-Ia*	*qnrS1*, *qnrs2,* *oqxA*, *oqxB*, *aac (6′)-Ib-cr*, *arr-3*	*mph(A)*, *mph(E)*, *msrE*, *tet(A)*, *tet(B)*, *sul1*, *sul2*, *dfrA12*, *dfrA17*, *dfrA5*, *floR*, *catA*, *catB3*, *fos*A	[26]
**China**	Adult and Pediatric	NR^1^	7	ST1193, ST448 ST361, ST410 ST405, ST167	*bla*_NDM-1_, _-5_	*bla*_TEM-1B_ *bla*_CTX-M-15_	*aadA2*, *rmtB*	NR^1^	*sul1*, *dfrA12*, *qacE*	[27]
**South Korea**	Adult and Pediatric	Outpatient department General ward, ICU^2^	12	NR^1^	*bla*_KPC-2, -__18_ *bla*_NDM-5_ *bla*_OXA-181_	*bla*_CTX-M-1, -9,_ _-15, -14, -25, -55_ *bla*_DHA-1_ *bla*_CMY-2_	NR^1^	NR^1^	NR^1^	[28]
**Japan**	Adult	NR^1^	3	ST131 ST2750	*bla*_IMP-6_	*bla*_CTX-M-2, -27_	NR^1^	NR^1^	NR^1^	[29]
**Japan**	Adult	Surgery, Neurosurgery, Orthopedics, Internal medicine	1	ST131	*bla*_IMP-6_	NR^1^	NR^1^	NR^1^	NR^1^	[30]
**India**	Pediatric	NR^1^	11	NR^1^	*bla*_NDM_ *bla*_OXA-48-like_	NR^1^	NR^1^	NR^1^	NR^1^	[31]
**Gabon**	Pediatric	Maternity and Neonatology	1	ST2083	*bla*_NDM-5_	*bla*_TEM_ *bla*_CMY_	*aac(6′)Ib-r*, *aac(3)-Ild*, *aadA5*, *aph(6)-Id, aph(3″)-Ib*	NR^1^	*drfA17*, *mph(A)*, *sul1*, *sul2*, *tetB*, *qacE*	[32]
**USA**	Pediatric	NR^1^	3	NR^1^	*bla*_NDM-5_	*bla*_EC-18_	NR^1^	NR^1^	*ftsl*, *acrD*, *ermA*, *barA*, *cirA*, *panF*, *artM*, *dadA*	[33]
**USA**	Adult and Pediatric	NR^1^	28	ST2, ST692, ST53, ST87,ST88, ST960, ST8, ST39, ST1017, ST477	*bla*_NDM-1,_ _-5_ *bla*_KPC-2_	NR^1^	NR^1^	NR^1^	NR^1^	[34]
**China**	Adult	NR^1^	1	ST410	*bla*_NDM-5_	*bla*_CTX-M-14_	*aph(3′)-Ia*, *aadA1*, *aadA2*, *ant(3″)-Iia*, *aac(6′)-Ib10*, *aac(6′)-Ib-cr*	*aac (6′)-Ib-cr*	*tetM*, *sul2*, *dfrA12,* *mdf(A)*, *cmlA*, *floR*, *qacH*, *fyuA*, *iutA*, *terC*, *iucC*, *afaABCDE*, *lpfA*	[35]

**NR^1^**, not reported; **ICU^2^**, intensive care unit; **NICU^3^** neonatal intensive care unit; **STs**, sequence types, **CPase genes**, Carbapenemase-encoding genes; **ESBL genes**, extended-spectrum β-lactamase-encoding genes; **Mac-R**, macrolide resistance; **Tet-R**, tetracycline resistance; **Sul-R**, sulfonamide resistance; **BSIs**, bloodstream infections.

**Table 2:** Studies reporting carbapenemase–plasmid associations in CP-Ec bloodstream isolates (BSIs), with STs, plasmid replicon type/size, and reference.

Country	No. of CP-Ec BSI Isolates	Cpase Genes	Plasmid Replicon (s)	Plasmid Size (bp)	ST (s)	Reference
**Germany**	2	*bla*_OXA-48_	IncF IncL	80,000 bp 63,000 bp	ST393 ST354	[19]
**China**	1	*bla*_NDM-3_	IncI1	NR^1^	ST977	[23]
**China**	1	*bla*_NDM-5_	IncX3	NR^1^	ST410	[24]
**China**	1	*bla*_NDM-5_	IncX3	~46,161 bp	ST354	[25]
**China**	8	*bla*_NDM-5_	IncX3 IncFII/IncI1 Multireplicon IncF (IncFIA/IncFIB/IncFII/Q1)	NR^1^	ST410, ST38, ST58, ST131, ST156, ST661	[26]
**China**	7	*bla*_NDM-1_	*bla*_NDM-1_ IncN	*bla*_NDM-1_ IncN (54,734 bp)	*bla*_NDM-1_ ST1193	[27]
		*bla*_NDM-5_	*bla*_NDM-5_ IncX3, IncFII, IncI	*bla*_NDM-5_ IncX3 (46,158 bp) IncX3 (48,787 bp) IncX3 (52,884 bp) IncFII (95,073 bp) IncFII (91,572 bp) IncI (26,828 bp)	ST448 ST361 ST410 ST405 ST405 ST167	
**Japan**	1	*bla*_IMP-6_	IncN	NR^1^	ST131	[30]
**China**	1	*bla*_NDM-5_	IncX3	NR^1^	ST410	[35]

**NR^1^**, not reported; **CPase genes**, Carbapenemase-encoding genes.

## Data Availability

No new data were created or analyzed in this study.
